# Quantitative EEG and its relationship with attentional control in patients with anxiety disorders

**DOI:** 10.3389/fpsyt.2024.1483433

**Published:** 2024-11-11

**Authors:** Danfeng Yuan, Xiangyun Yang, Pengchong Wang, Lijuan Yang, Ting Yang, Fang He, Yi Xu, Zhanjiang Li

**Affiliations:** ^1^ Beijing Key Laboratory of Mental Disorders, National Clinical Research Center for Mental Disorders, Beijing Anding Hospital, Capital Medical University, Beijing, China; ^2^ Advanced Innovation Center for Human Brain Protection, Capital Medical University, Beijing, China

**Keywords:** anxiety disorders, beta power, theta beta ratio, attentional control, resting-state EEG

## Abstract

**Introduction:**

Attentional control is crucial in the development and maintenance of anxiety disorders. Understanding the underlying mechanisms of attentional control can help to shed light on the neuropathological processes in anxiety disorders (ANX). Quantitative electroencephalography (QEEG) offers a cost-effective, noninvasive method for examining the neuropathological mechanisms of mental disorders.

**Methods:**

In this study, 67 patients with ANX and 45 healthy controls (HC) were recruited. EEG recordings were obtained for 5 minutes in an eyes-closed condition. QEEG was employed to evaluate the mechanisms of attentional control in ANX.

**Results:**

Neurophysiological measures indicated that anxiety patients exhibited a more frontal topographic pattern of theta/beta ratio (TBR) compared to HC. Additionally, a significant decrease in temporal beta power was observed in the ANX group. Correlation analysis revealed that decreased beta power and increased TBR were significant association between attentional control deficits in ANX.

**Discussion:**

These findings provide electrophysiological evidence of impaired attentional control processing in anxiety patients, characterized by decreased temporal beta power and increased frontal TBR. Temporal beta power and frontal TBR may serve as promising biomarkers for attentional control in ANX.

## 1 Introduction

Anxiety disorders (ANX) are the most prevalent group of mental disorders, characterized by high prevalence, chronicity and comorbidity (1). As a spectrum disorder, the core symptoms of ANX are fear and anxiety. Additionally, patients with ANX often exhibit cognitive deficits, especially in attentional control (2). However, the neurobiological mechanisms underlying anxiety disorders are not well understood. Attentional control refers to the ability to direct attention toward or away from stimuli based on current goals or task demands (3). Poor attentional control in ANX, an important transdiagnostic symptom, can reduce coping abilities and impair social and occupational functioning (4). Attentional control theory (ACT) indicates that impaired attentional control is related to the onset and maintenance of anxiety (5). Thus, exploring the neural mechanisms of attentional control in ANX population paves the way for a better understanding of the neurobiological mechanisms of ANX.

Electroencephalography (EEG) is a noninvasive and inexpensive technique for measuring cortical electrical activity with high temporal resolution (6). In the previous studies, event-related potentials (ERPs) (error-related negativity (ERN) and N2) are used extensively to investigated the neural mechanisms of attentional control in ANX (7), which suggested that individuals with ANX exhibited significant higher N2 and ERN amplitudes due to impaired attentional control (8, 9). However, ERPs related studies of attentional control mechanisms in ANX are typically task-induced. Previous neuropsychological studies demonstrated that the attentional control difficulties are not specific to stimuli but reflect a broader dysregulation, even when stimuli are absent (10). For example, a brain imaging study at rest demonstrated that individuals with high anxiety exhibited lower functional connectivity between the anterior cingulate cortex (ACC) and superior frontal gyrus (IPG). The general prefrontal control deficit may be involved in the pathophysiology of attentional control in anxiety (11). Examining the potential electrophysiological indicators related to attentional control during resting state can provide important supplementary insights into the neural basis of attentional control deficits in individuals with ANX.

Quantitative electroencephalography (QEEG) technique, which could capture the background brain activity, has been used as a supplementary tool to complete the visual analysis like ERPs. Evidence has presented that QEEG has been widely used in the studies of neurological and psychiatric disorders (12, 13). The most common QEEG analysis is the assessment of the absolute and relative spectral power in specific frequency bands. Recently, increasing interest in the ratio of spectral power between slow and fast bands suggests that these EEG ratios can amplify subtle abnormalities and reflect complex cognitive states (14).

Several studies have investigated potential QEEG markers for attentional control in non-ANX samples. QEEG findings suggested that the theta/beta ratio (TBR) might be the most promising indicator of attentional control (15). Additionally, a decrease in the activities in high-frequencies (alpha and beta) have been extensively reported that might be the neural correlate of cognitive decline in patients with Attention-Deficit Hyperactivity Disorder (ADHD) and major depressive disorder (MDD) (16, 17). ADHD and MDD are often accompanied by significant anxiety symptoms, particularly with ADHD and MDD sharing a high comorbidity with ANX (18, 19). Considering the emphasis and importance of attentional control theory in understanding the relationship between anxiety and attentional control, the QEEG features may reflect the electrophysiological basis of how anxiety affects attentional control in patients with ADHD, MDD, and other disorders during non-task states. This also raises the question of whether the increased TBR and decreased power of higher frequency bands are associated with attentional control deficits in the ANX population during resting state due to high levels of anxiety. However, no previous studies have explored the association between attentional control and different patterns of spontaneous EEG measures in clinical anxiety patients.

Based on the previous neuropsychological literature and ACT, the purpose of the current study is to assess the QEEG characteristics of anxiety patients. In addition, we aim to explore the relationships between self-reported attentional control and the aforementioned QEEG measures to find a potential biomarker for attentional control in ANX.

## 2 Materials and methods

### 2.1 Participants

The anxiety group in this study consisted of outpatients from Beijing Anding Hospital. The inclusion criteria were as follows: (1) a primary diagnosis of anxiety disorders by an experienced psychiatrist according to the Diagnostic and Statistical Manual of Mental Disorders, 5th edition (DSM-5), (2) aged between 18 and 60 years, (3) drug-naive or psychotropic drug-free for at least four weeks, (4) scores less than 17 on the 17-item version of the Hamilton Depression Rating Scale (HAMD-17), and (5) right-handedness. The exclusion criteria were: (1) a diagnosis of schizoaffective disorder, (2) neurological disorder, (3) significant head injury, (4) suicidal risk, or (5) current substance use (tobacco, alcohol, and stimulants). The control group consisted of age, sex, and education-matched healthy subjects. In the control group, subjects with a history of any psychiatric illness, major physical illness, neurological illness, significant head injury, family history of psychiatric illness in first-degree relatives or current use of substances were excluded. All participants were right-handed and provided informed consent after a complete description of the study. The study design was approved by the Ethics Committee of Beijing Anding Hospital, affiliated with Capital Medical University (202395FS-2).

### 2.2 Questionnaire

Questionnaires were administered before the resting-state EEG recording. The severity of anxiety symptoms was assessed using Zung’s Self-Rating Anxiety Scale (SAS) and the State-Trait Anxiety Inventory (STAI). The SAS is a widely utilized norm-referenced scale that screens for anxiety disorders based on psychological symptoms from the past week (20). The cut-off score for clinically significant anxiety is set at 45 (21). The STAI is a well-known and reliable self-report scale for measuring anxiety, consisting of 40 items, with 20 items allocated to each of the state anxiety and trait anxiety subscales. A score of ≥40 is considered clinically significant (22). The severity of depression symptoms was assessed using the 17-item Hamilton Rating Scale for Depression (HAMD-17), one of the most common observer-rated scales for evaluating depression symptoms (23). Attentional control was assessed using the Attentional Control Scale (ACS), a self-report questionnaire developed to measure individual differences in attentional control, which includes two factors: focusing and shifting (24). The Chinese version of the ACS was modified in 2020 and demonstrated good validity and reliability (25).

### 2.3 Procedure

During the EEG recording, participants were seated comfortably in a quiet, darkened room. Five minutes of spontaneous EEG data were recorded in an eyes-closed condition (EC). EEG activity was continuously recorded using a 64-electrode cap (ActiCap64 system, Brain Products GmbH, Gilching, Germany) according to the International 10-20 system. Impedance was kept below 10 kΩ. Reference electrodes were placed on the bilateral mastoids. EEG data were bandpass filtered between 1-40 Hz, with the notch frequency set to 48-52 Hz. Data were visually inspected first to remove electrodes with high impedance or noisy signals. The bad channels were interpolated by surrounding channels. Ocular artifacts from eye blinks and horizontal eye movements were corrected using independent component analysis. The continuous EEG data were segmented into 2000-ms epochs. Epochs with amplitudes exceeding ±100 μV were marked as artifacts and automatically removed. Data were also visually inspected for any remaining artifacts.

### 2.4 Power spectral analysis

Absolute power was computed using a Fast-Fourier Transformation with a 50% Hanning window over each epoch in four frequency bands: delta (1-4 Hz), theta (4–8 Hz), alpha (8-12 Hz), and beta (12–30 Hz). Relative spectral power was calculated as the ratio of the power at that frequency to the total power spectrum across all frequencies of interest (1-30 Hz). The EEG ratio was calculated by dividing slow band (delta, theta) power by fast band (alpha, beta) power. EEG powers across all frequencies were extracted from five brain regions: frontal (Fp1, Fp2, F3, F4, F7, F8, Fz, FC1, FC2, FC5, FC6, F1, F2, AF3, AF4, FC3, FC4, F5, F6, FT7, FT8, FPz), temporal (T7, T8, TP7, TP8), central (C3, C4, Cz, CP1, CP2, CP5, CP6, C1, C2, CP3, CP4, C5, C6, CPz), parietal (P3, P4, P7, P8, Pz, P1, P2, PO3, PO4, P5, P6, PO7, PO8, POz), and occipital (O1, O2, Oz).

### 2.5 Statistical analysis

All data were analyzed using Statistical Products and Services Solutions (SPSS, version 26.0). The Chi-square test was performed for categorical variables, and the two-tailed independent *t*-test was used to compare continuous demographic information between individuals with ANX and healthy controls. To compare the power spectral features between groups, repeated-measures analysis of variance (ANOVA) was conducted for each frequency band. Pairwise multiple comparisons were further conducted to compare group differences in spectral power across different frequency bands and regions. The EEG spectral characteristics that showed significant differences between patients with ANX and healthy controls were analyzed in a correlation analysis. Pearson correlation analysis was performed to assess the correlation between QEEG features and self-reported attentional control in ANX. Statistical significance was set at *p*< 0.05 (two-tailed).

## 3 Results

### 3.1 Demographics and clinical characteristics

A total of 67 patients with ANX (44 women, mean age 33.05 (8.34), range 20-55) and 45 healthy controls (35 women, mean age 31.24 (12.83), range 22-59) were included in the present study. The demographic and clinical features are presented in **Table 1**. No significant difference in age, sex, and education was detected between subjects with ANX group and healthy controls. Of 67 patients with ANX included in the study, 31 were diagnosed as panic disorder (PD), 27 were diagnosed as generalized anxiety disorder (GAD), and 9 were diagnosed as social anxiety disorder (SAD). Five had a comorbidity: one patient with GAD comorbid SAD (n=1), two patients with PD comorbid post-traumatic stress disorder (PTSD) (n=2). Two patients with PD comorbid GAD (n=2). Regarding clinical characteristics, the mean SAS and STAI scores of the anxious group were 47.48 and 98.14, respectively, both exceeding the cut-off score for ANX (SD = 10.90; SD = 17.99). The mean HAM-D score of the anxiety group was 10.72 that falls within the range of mild depression (SD = 3.94). For evaluation of attentional control, the mean ACS score of anxiety group was 48.13 that significantly lower than the healthy controls.

**Table 1:** Demographic characteristics and baseline clinical characteristics of all the participants.

Variables	ANX	Controls	t/χ2	*p*
Age (years)	33.05 (8.34)	31.24 (12.83)	0.892	0.374
Gender (M/F)	23/44	10/35	1.898	0.168
Education (years)	13.1 (2.0)	12.7 (2.8)	0.7	0.488
Disease course (months)	16.5 (6.8-36.0)	–	–	–
HAM-D	10.72 (3.94)	1.278 (1.19)	14.554	0.000
SAS	47.48 (10.90)	34.00 (6.34)	7.035	0.000
STAI	98.14 (17.99)	66.10 (19.60)	7.959	0.000
ACS-AF	27.56 (8.18)	31.15 (5.60)	-2.355	0.021
ACS-AS	20.58 (4.63)	27.87 (6.25)	-6.566	0.000
ACS	48.13 (9.71)	56.15 (15.59)	-3.079	0.003

Values are expressed as the mean ± SD or median (25%–75% quartile); M, Male; F, Female; ACS, Attention Control Scale; ACS-AF, Attention Focusing Subscale; ACS-AS, Attention Shifting Subscale; HAM-D, Hamilton Depression Scale; SAS, Self-Rating Anxiety Scale; STAI, State-Trait Anxiety Inventory; ANX, anxiety disorders; HC, healthy controls.

### 3.2 Spectrum power differences between ANX and HC

**Figure 1:**
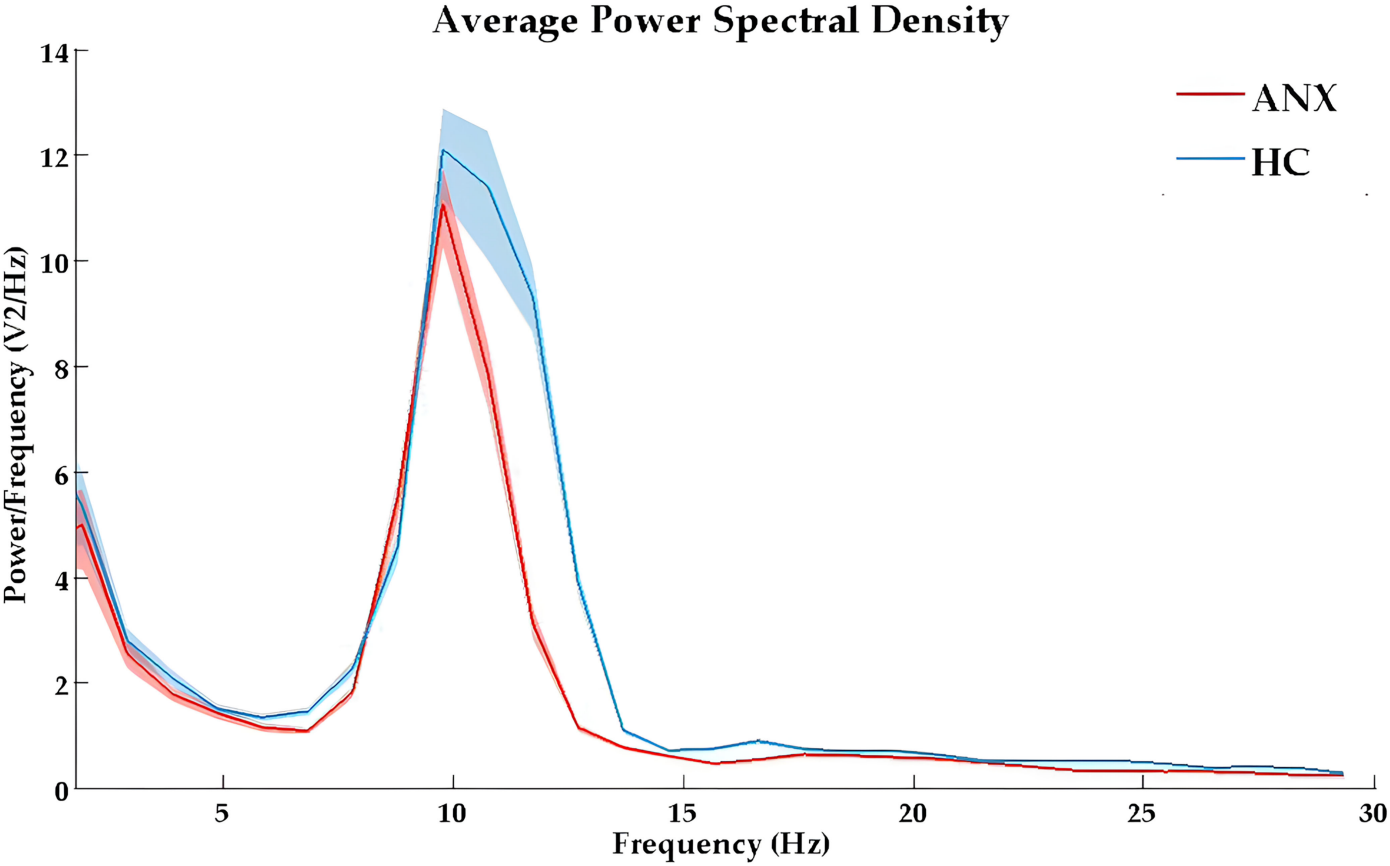
The absolute power spectral density curves in eyes-closed condition for ANX (red) and healthy control (blue) groups. ANX, anxiety disorders; HC, healthy controls.

The full power spectrum for both groups under eyes-closed conditions at average channel locations is shown in **Figure 1**. The topographical maps of absolute power and relative power in anxiety and healthy control groups are presented in **Supplementary Figures 1**, **2**. Compared to the healthy controls, the anxiety group exhibited a global decrease in spectral power at fast frequencies and an increase in spectral power at slower frequency bands.

**Figure 2:**
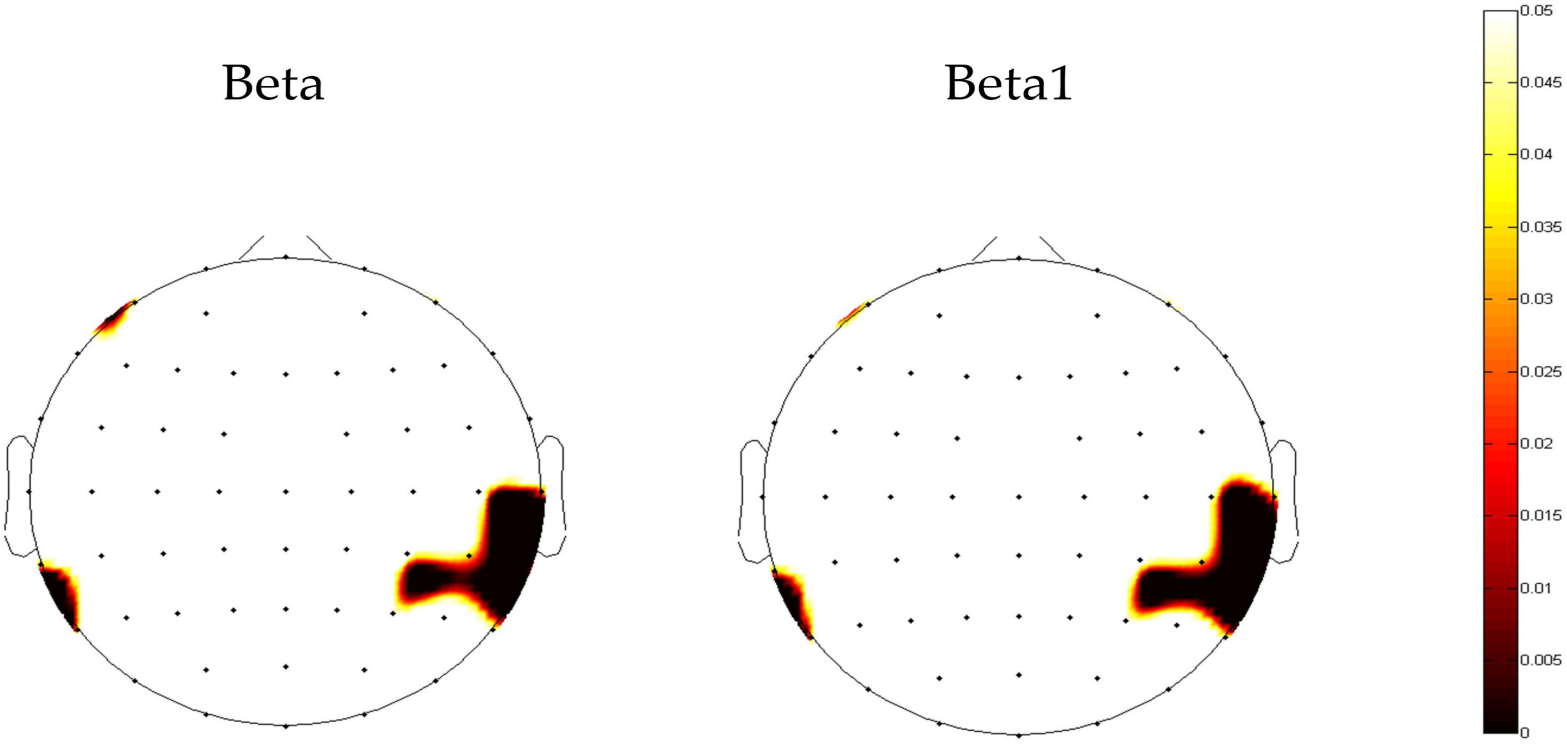
The topography map of *p*-value in beta power between anxiety disorders and control groups. Red areas represent significant differences (*p*<0.05).

The results of the repeated measures ANOVA for each frequency band are presented in **Table 2**. A significant main effect of group was observed only in absolute beta power (*F* = 4.068, *p* = 0.046, ηp² = 0.036). As beta oscillations can be roughly subdivided into the low-beta band (12-20 Hz) and high-beta band (20-30 Hz), we further compared the absolute and relative spectral power in the low-beta and high-beta frequency bands between the two groups. The average beta power in each region in both groups is presented in **Table 3**. The independent *t*-test analysis revealed that a significant difference between the two groups in absolute beta power was found in temporal regions (*p* = 0.015) (**Figure 2**). Patients with ANX showed significantly lower beta band power in the temporal regions compared to healthy controls. Additionally, a significant group difference was observed in low-beta power in the temporal regions (*p* = 0.018) (**Figure 2**), with patients with ANX exhibiting attenuated low-beta power compared to healthy controls.

**Table 2:** Repeated measure ANOVA of absolute powers, relative powers, and spectral power ratios between ANX and control groups.

Variables	*F*	*p*	η^2^
Delta			
Group	2.249	0.137	0.020
Group×Region	1.524	0.219	0.014
Theta			
Group	0.037	0.848	0.000
Group×Region	2.816	0.059	0.025
Alpha			
Group	0.763	0.384	0.007
Group×Region	1.384	0.252	0.012
Beta			
Group	4.068	**0.046**	0.036
Group×Region	0.128	0.897	0.001
Relative delta			
Group	0.361	0.549	0.003
Group×Region	1.262	0.286	0.011
Relative theta			
Group	0.092	0.762	0.001
Group ×Region	1.015	0.355	0.009
Relative alpha			
Group	1.075	0.302	0.010
Group ×Region	0.281	0.698	0.003
Relative beta			
Group	0.839	0.362	0.008
Group ×Region	0.201	0.829	0.002
TBR			
Group	0.288	0.593	0.003
Group ×Region	3.379	**0.039**	0.030
TAR			
Group	0.035	0.852	0.000
Group ×Region	0.397	0.628	0.004
DBR			
Group	0.180	0.672	0.002
Group ×Region	0.218	0.741	0.002
DAR			
Group	0.201	0.654	0.002
Group ×Region	0.157	0.741	0.001

TBR, theta/beta ratio; TAR, theta/alpha ratio; DBR, delta/beta ratio; DAR, delta/alpha ratio. Bold values denote statistical significance at the p<0.05 level.

**Table 3:** Mean beta powers by region for ANX and HC groups.

Variables	ANX	Controls	t/χ2	*p*
Frontal region				
Beta	0.542 (0.260)	0.646 (0.432)	-1.581	0.117
Beta1	0.758 (0.392)	0.914 (0.739)	-1.450	0.150
Beta2	0.407 (0.213)	0.431 (0.251)	-0.537	0.593
Temporal region				
Beta	0.289 (0.128)	0.420 (0.334)	-2.520	**0.015**
Beta1	0.398 (0.194)	0.599 (0.530)	-2.444	**0.018**
Beta2	0.223 (0.116)	0.277 (0.203)	-1.616	0.111
Central region				
Beta	2.293 (0.983)	2.607 (1.352)	-1.562	0.123
Beta1	0.727 (0.465)	0.913 (0.945)	-1.498	0.137
Beta2	0.349 (0.208)	0.394 (0.278)	-0.992	0.324
Parietal region				
Beta	1.984 (0.908)	2.305 (1.212)	-1.612	0.112
Beta1	0.812 (0.529)	1.027 (0.811)	-1.569	0.121
Beta2	0.370 (0.240)	0.399 (0.283)	-0.582	0.562
Occipital region				
Beta	1.885 (0.959)	2.334 (1.388)	-1.561	0.121
Beta1	0.826 (0.631)	1.036 (0.820)	-1.282	0.203
Beta2	0.402 (0.236)	0.433 (0.310)	-0.607	0.545

Values are expressed as the mean ± SD; ANX, anxiety disorders; HC, healthy controls. Bold values denote statistical significance at the *p*<0.05 level.

### 3.3 Spectral power ratios differences between ANX and HC

**Figure 3:**
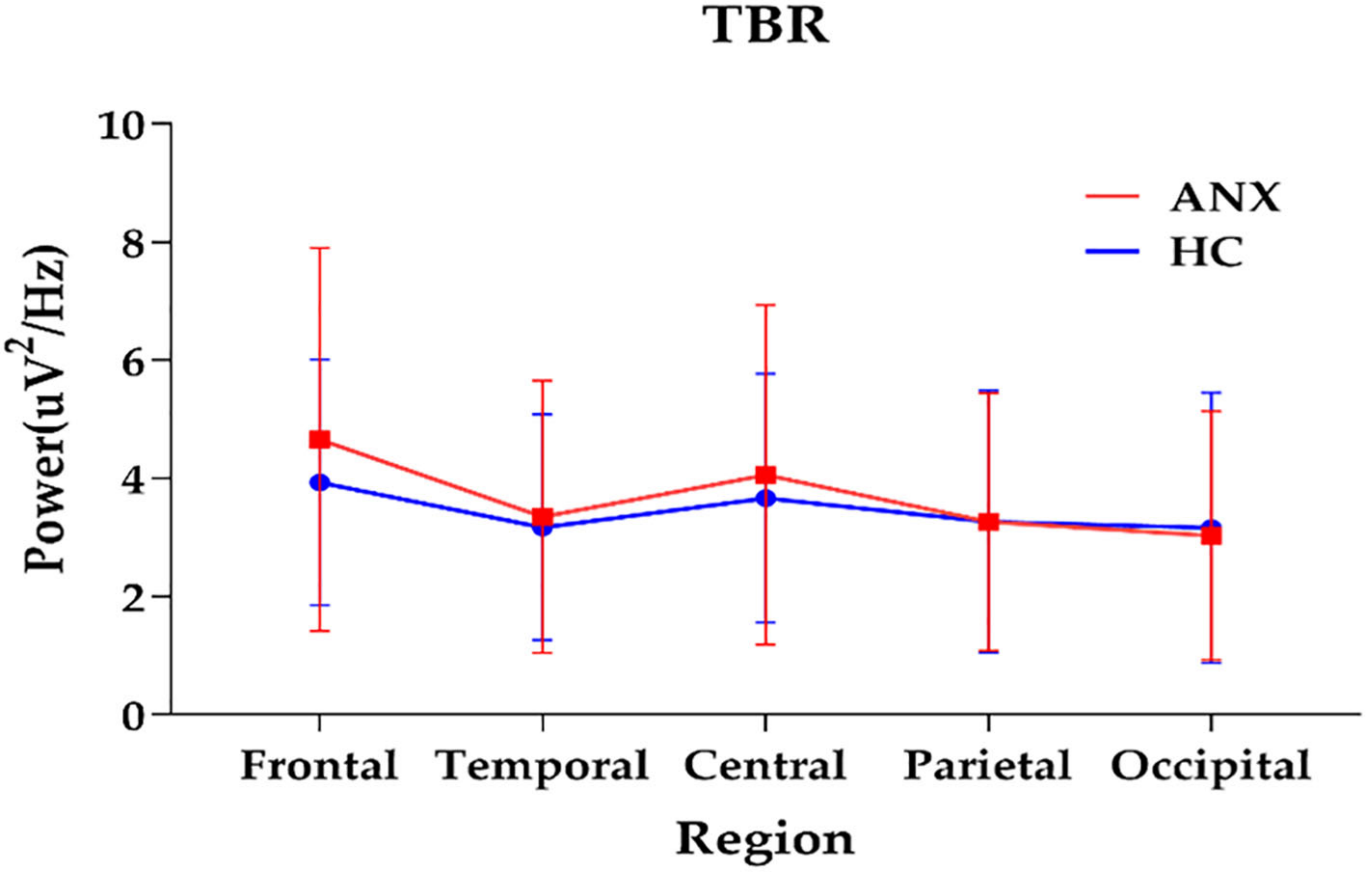
The interactions between group and region on TBR (plot: mean with SD). ANX, anxiety disorders; HC, healthy controls; TBR, theta/beta ratio.

The topographical maps of the slow to fast spectral power ratio in anxiety and healthy control groups were presented in **Supplementary Figure 3**. The anxiety group exhibited an overall increase in slow-to-fast power ratios. The results of repeated measures ANOVA showed significant interactions between group and region in TBR (*F* = 3.379, *p*= 0.039, ηp² = 0.030) (**Table 2**). A different topographic distribution of TBR in the ANX group was observed (**Figure 3**). Changes in TBR across the five brain regions were more pronounced in the ANX group than in healthy controls. Additionally, the TBR were numerically higher in the anxious group at frontal-central regions but lower at parietal-occipital regions compared with healthy controls. However, no significant main effect of group was found in any slow-to-fast power ratios.

### 3.4 Spectrum power differences between PD and GAD

To explore the difference in EEG spectral characteristics in different subtypes of ANX, the independent *t*-test between patients with PD and GAD was conducted. However, the results showed no significant differences between patients with PD and GAD in absolute powers, relative powers, and spectral power ratios at five brain regions (**Supplementary Figure 4**).

### 3.5 Association between spectral power and attentional control

**Figure 4:**
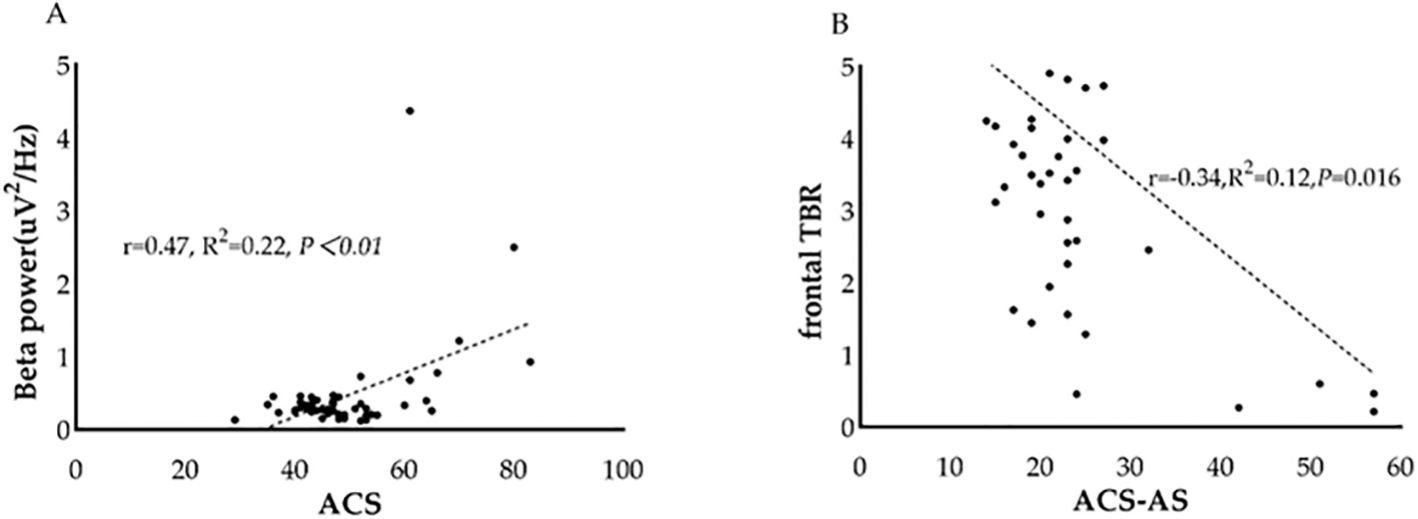
Association between QEEG index and attentional control scores in patients with ANX. **(A)** The relationship between beta power and attentional control scores in patients with ANX. **(B)** The relationship between frontal theta/beta ratio and attentional shifting scores in patients with ANX. ACS, attentional control scores; TBR, theta/beta ratio.

Given the observed group difference in EEG spectral power between ANX patients and healthy controls, specifically in absolute beta power at the temporal region and TBR, we further investigated the relationship between these differentiated QEEG indices and self-reported attentional control in patients with ANX. We found that, in patients with ANX, attentional control scores assessed by ACS were found to be positively correlated with absolute beta power in the temporal region (r= 0.470, *p* < 0.01). In addition, a negative correlation was also found between attentional shifting and frontal TBR in patients with ANX (r= -0.355, *p*= 0.011) (**Figure 4**).

## 4 Discussion

In this study, we confirmed deficits of attentional control in anxiety patients. QEEG analysis revealed an aberrant topographic pattern of TBR and a significantly attenuated beta power in patients with ANX. Correlation analysis indicated that beta power at temporal region and frontal TBR might be potential electrophysiological index of attentional control in patients with ANX.

The spectral power analysis showed that patients with ANX displayed lower beta power in the temporal regions. Generally, beta power in different frequency ranges is related to different functional roles. Low-beta wave activity is more likely associated with sustained attention, perception of the environment, and working memory, while high-beta might reflect anxiety, muscular tension, or anger (26). We further compared the beta power of the two groups in the low-beta and high-beta bands. The results indicated that patients with ANX exhibited attenuated beta power only in the low-beta frequency, not in the high-beta bands. Three QEEG studies have reported potential associations between aberrant absolute beta power and anxiety (27–29). Byeon et al. suggested that patients with higher beta and high-beta waves in T3 and T4 are more likely to respond to medication in anxiety disorders (29). The more severe the anxiety symptoms, the higher the probability of response to pharmacological treatment (30), consistent with our findings that patients with ANX are more likely to exhibit decreased beta power in temporal regions.

Previous quantitative EEG studies only explored differences in spectral powers between ANX and healthy controls from a broad category. However, there is a lack of studies comparing the characteristics of quantitative EEG among different subtypes of ANX. In this study, we investigated the difference in the powers of spectral activities between patients with PD and GAD. However, no significant difference was detected between groups of GAD and PD. The result suggested that the aberrant spectral power features in beta band might be a common alternation in patients with ANX. It might be difficult to distinguish specific subtypes of ANX by QEEG features alone.

Another interesting finding in our study was that the group difference in beta power was mainly on the right side of the brain, indicating decreased beta power in the right temporal lobe in patients with ANX. Previous studies also reported that left-sided behavior bias is associated with symptoms of anxiety, fear, anhedonia, behavioral despair, and stress exposure (31). This result supports the association between affective disorders and abnormal lateralization of brain function (32).

The slow-to-fast EEG ratio could reflect the cortical-subcortical interactions, which is implement by the prefrontal cortex (33). Higher ratio scores may indicate reduced cortical control function over subcortical drives. Increased frontal TBR was previously reported in individuals with high trait anxiety (34, 35). The results of EEG ratios in our study found a significant interaction of Group×Region on TBR, especially a significant increase in frontal TBR. This finding suggests that patients with ANX exhibited a more frontal topographic distribution of TBR. Our study corroborates previous findings that participants with high anxiety levels show elevated TBR in clinical anxiety patients.

Taken together, the results of EEG spectral features and EEG ratios suggest that patients with ANX show an increase in low-frequency power and a decrease in fast-frequency power, indicating a possible deficiency in top-down attention control in anxiety. Among these findings, the most significant difference between patients with ANX and healthy controls was beta power in the temporal regions.

The correlation analysis suggested that temporal beta power was positively associated with attentional control scores assessed by ACS in patients with ANX. Some ERP components, such as N2 or ERN, have been found to reflect attentional control dysfunction (36). In addition to ERP components, frontal TBR is considered a potential electrophysiological marker of attentional control in anxiety (37). To our knowledge, this is the first study to find a correlation between beta activity in temporal regions and attentional control in individuals with ANX. Beta oscillation has been found be associated with attentional top-down modulation (37). Decreased beta band activity might reflect a transition towards a stimulus-driven state and cognitive impairment, which is consistent to the neural mechanism of attentional control deficits in non-ANX sample (38, 39). For example, a study reported the correlation between symptoms of inattention and beta power in MDD (40). Research on ADHD also reported the association between aberrant parietal and frontal beta rhythmic and working memory deficits (41). Neuroimaging evidence suggested that the bilateral temporoparietal junction plays a critical role in processing bottom-up information for top-down control of attention, which is consistent to our finding in temporal region (42). The correlation between frontal TBR and attentional shifting ability in ANX was found to be significant. This result confirmed the previous findings that increased TBR is associated with impaired attentional control (43). More specifically, high levels of anxiety might induce difficulty in shifting performance and exhibit high TBR in rest state.

In summary, we suggested that the attentional control deficits in anxiety patients not only exhibited in task-related paradigms but also in task-free period. In addition, beta band power and frontal TBR may serve as potential indicators of attentional control in clinical anxiety patients.

This study has several limitations. First, we conducted only a cross-sectional study and did not perform any follow-up. Additionally, we only measured EEG oscillations at task-free condition. Future studies could incorporate task-related EEG parameters to better explore the neural mechanisms of attentional control in ANX. Third, the sample was heterogeneous. Although we performed a subgroup analysis, we only compared the QEEG features of two common anxiety subtypes: PD and GAD. However, other subtypes, such as SAD, were not investigated separately due to an insufficient sample size. Finally, the attentional control ability was only measured by a self-reported questionnaire. More objective assessment tools of cognitive function, like neuropsychological tests, could be incorporated in the future study to improve objectivity of the measurement.

## 5 Conclusion

In conclusion, the present study investigated the differences in spectral power and slow/fast frequency power ratio between ANX patients and healthy controls. Our findings provided electrophysiological evidence of attentional control deficits in ANX, characterized by a decrease in absolute beta power in the temporal regions and a more frontal topographic distribution of TBR. Additionally, our findings suggest that temporal beta power and frontal TBR might be promising markers of attentional control in ANX.

## Data Availability

The raw data supporting the conclusions of this article will be made available by the authors, without undue reservation.
