# Supplementary material for: Quantitative EEG and its relationship with attentional control in patients with anxiety disorders
Source: Front Psychiatry. 2024 Nov 11;15:1483433. doi: 10.3389/fpsyt.2024.1483433 (PMC11586352; doi:10.3389/fpsyt.2024.1483433)
Supplement: Supplementary file 1 [file DataSheet1.docx]

Supplementary Material

**Supplementary Figure 1.** Topographical maps of absolute power in anxiety disorders and healthy control groups. Red represents higher values and blue represents lower values. Abbreviations: ANX: anxiety disorders; HC: healthy controls

**Supplementary Figure 2.** Topographical maps of relative power in anxiety disorders and healthy control groups. Red represents higher values and blue represents lower values. Abbreviations: ANX: anxiety disorders; HC: healthy controls

**Supplementary Figure 3.** Topographical maps of spectral power ratio in anxiety disorders and healthy control groups. Red represents higher values and blue represents lower values.

Abbreviations: ANX: anxiety disorders; HC: healthy controls; TAR:theta/alpha ratio; TBR:theta/beta ratio; DAR: delta/alpha ratio; DBR: delta/beta ratio
