# Supplementary material for: Quantitative EEG and its relationship with attentional control in patients with anxiety disorders
Source: Front Psychiatry. 2024 Nov 11;15:1483433. doi: 10.3389/fpsyt.2024.1483433 (PMC11586352; doi:10.3389/fpsyt.2024.1483433)
Supplement: Supplementary file 2 [file Table1.docx]

**Supplementary Table 1.** Comparison of absolute and relative powers between panic disorder and generalized anxiety disorder.

| **Variables** | **PD(n=31)** | **GAD(n=27)** | ***t*** | ***p*** |
| --- | --- | --- | --- | --- |
| Frontal region |  |  |  |  |
| Delta | 4.004(1.366) | 3.496(1.277) | 0.773 | 0.443 |
| Theta | 2.581(2.061) | 2.618(1.325) | -0.055 | 0.956 |
| Alpha | 7.070(3.906) | 6.967(3.791) | 0.060 | 0.953 |
| Beta | 0.583(0.254) | 0.514(0.210) | 0.982 | 0.330 |
| Relative delta | 0.338(0.128) | 0.330(0.105) | 0.181 | 0.857 |
| Relative theta | 0.177(0.067) | 0.185(0.062) | 0.096 | 0.924 |
| Relative alpha | 0.450(0.123) | 0.467(0.156) | -0.305 | 0.762 |
| Relative beta | 0.052(0.027) | 0.049(0.031) | 0.390 | 0698 |
| Temporal region |  |  |  |  |
| Delta | 1.429(0.545) | 1.405(0.619) | 0.124 | 0.902 |
| Theta | 1.006(0.815) | 0.989(0.511) | 0.063 | 0.949 |
| Alpha | 2.350(1.244) | 2.537(1.365) | -0.357 | 0.723 |
| Beta | 0.309(0.176) | 0.276(0.256) | 0.938 | 0.352 |
| Relative delta | 0.330(0.136) | 0.346(0.105) | -0.418 | 0.678 |
| Relative theta | 0.181(0.070) | 0.180(0.053) | 0.096 | 0.924 |
| Relative alpha | 0.426(0.120) | 0.455(0.159) | -0.513 | 0.610 |
| Relative beta | 0.075(0.059) | 0.063(0.047) | 1.283 | 0.205 |
| Central region |  |  |  |  |
| Delta | 2.518(1.083) | 2.423(0.924) | 0.232 | 0.817 |
| Theta | 2.028(1.926) | 2.173(1.242) | -0.243 | 0.809 |
| Alpha | 5.945(3.670) | 6.187(3.749) | -0.151 | 0.880 |
| Beta | 0.537(0.272) | 0.479(0.247) | 0.756 | 0.453 |
| Relative delta | 0.288(0.115) | 0.302(0.166) | -0.358 | 0.722 |
| Relative theta | 0.178(0.056) | 0.189(0.066) | -0.640 | 0.525 |
| Relative alpha | 0.485(0.144) | 0.477(0.142) | 0.158 | 0.875 |
| Relative beta | 0.060(0.033) | 0.053(0.031) | 0.900 | 0.372 |
| Parietal region |  |  |  |  |
| Delta | 2.054(0.789) | 2.239(1.071) | -0.516 | 0.608 |
| Theta | 1.598(1.029) | 1.999(1.112) | -0.902 | 0.371 |
| Alpha | 7.401(4.079) | 7.728(5.398) | -0.150 | 0.882 |
| Beta | 0.571(0.219) | 0.521(0.328) | 0.582 | 0.562 |
| Relative delta | 0.238(0.120) | 0.266(0.105) | -0.718 | 0.476 |
| Relative theta | 0.150(0.041) | 0.171(0.067) | -1.056 | 0.295 |
| Relative alpha | 0.560(0.169) | 0.498(0.154) | 1.235 | 0.222 |
| Relative beta | 0.066(0.043) | 0.054(0.028) | 1.166 | 0.248 |
| Occipital region |  |  |  |  |
| Delta | 2.011(0.807) | 2.132(1.107) | -0.338 | 0.736 |
| Theta | 1.543(0.803) | 1.899(1.049) | -0.877 | 0.384 |
| Alpha | 8.225(5.505) | 7.224(4.467) | 0.418 | 0.678 |
| Beta | 0.621(0.262) | 0.549(0.253) | 0.688 | 0.494 |
| Relative delta | 0.228(0.110) | 0.258(0.122) | -0.775 | 0.442 |
| Relative theta | 0.146(0.039) | 0.170(0.073) | -1.105 | 0.274 |
| Relative alpha | 0.569(0.155) | 0.501(0.167) | 1.312 | 0.195 |
| Relative beta | 0.072(0.040) | 0.057(0.032) | 1.368 | 0.177 |

PD: Panic disorder; GAD: Generalized anxiety disorder
